# Low filling ratio of the distal nail segment to the medullary canal is a risk factor for loss of anteromedial cortical support: a case control study

**DOI:** 10.1186/s13018-022-02921-z

**Published:** 2022-01-15

**Authors:** Hui Song, Shi-Min Chang, Sun-Jun Hu, Shou-Chao Du

**Affiliations:** Department of Orthopaedic Surgery, Yangpu Hospital, School of Medicine, Tongji University, 450 Tengyue Road, Shanghai, 200090 China

**Keywords:** Trochanteric fracture, Cephalomedullary nailing, Anteromedial cortical support, Filling ratio, Distal nail diameter, Medullary canal, Pendulum-like movement

## Abstract

**Background:**

Anteromedial cortical support apposition (positive and/or neutral cortical relations) is crucial for surgical stability reconstruction in the treatment of trochanteric femur fractures. However, the loss of fracture reduction is frequent in follow-ups after cephalomedullary nail fixation. This paper aimed to investigate the possible predictive risk factors for postoperative loss of anteromedial cortex buttress after nail fixation.

**Methods:**

A retrospective analysis of 122 patients with AO/OTA 31A1 and A2 trochanteric femur fractures treated with cephalomedullary nails between January 2017 and December 2019 was performed. The patients were classified into two groups according to the postoperative status of the anteromedial cortical apposition in 3D CT images: Group 1 with contact “yes” (positive or anatomic) and Group 2 with contact “No” (negative, loss of contact). The fracture reduction quality score, tip-apex distance (TAD), calcar-referenced TAD (Cal-TAD), Parker ratio, neck-shaft angle (NSA), and the filling ratio of the distal nail segment to medullary canal diameter in anteroposterior (AP) and lateral fluoroscopies (taken immediately after the operation) were examined in univariate and multivariate analyses. Mechanical complications were measured and compared in follow-up radiographs.

**Results:**

According to the postoperative 3D CT, 84 individuals (69%) were categorized into Group 1, and 38 individuals (31%) were classified as Group 2. The multivariate logistic regression analysis showed that the poor fracture reduction quality score (*P* < 0.001) and decreasing filling ratio in the lateral view (*P* < 0.001) were significant risk factors for the loss of anteromedial cortical contact. The threshold value for the distal nail filling ratio in lateral fluoroscopy predicting fracture reduction re-displacement was found to be 53%, with 89.3% sensitivity and 78.9% specificity. The mechanical complication (varus and over lateral sliding) rate was higher in Group 2.

**Conclusions:**

The fracture reduction quality score and the decreasing filling ratio of the distal nail to the medullary canal in the lateral view (a novel parameter causing pendulum-like movement of the nail) were possible risk factors for postoperative loss of anteromedial cortical support.

## Background

Currently, trochanteric hip fractures (AO/OTA 31A) are primarily treated using cephalomedullary nails [1–3]. However, postoperative complications and mechanical failures are frequent, ranging from 3 to 22%[4–6], with complications including over collapse of the head–neck fragment with femoral neck shortening or varus, helical blade/lag screw lateral back-out, cut-out from the superior femoral head, cut-in through the central femoral head, fracture non-union, and implant nail breakage.

In 1980, Kaufer [7] proposed five factors that can predispose fixation complications and treatment failure: bone quality, fracture morphology, implant selection, implant placement, and, most importantly, fracture reduction quality. In 2015, Chang et al. [8] proposed the concept of anteromedial cortex-to-cortex support reduction to obtain secondary stability after limited telescoping. The apposition of anteromedial cortices between the head–neck and shaft was assessed by immediate postoperative fluoroscopy in both AP and lateral views. This factor was classified as exhibiting positive, neutral, and negative patterns [8]. In practice, positive and neutral cortical appositions are acceptable; however, none of the negative relationships in the AP or lateral view was acceptable before nailing [9–11].

**Fig. 1:**
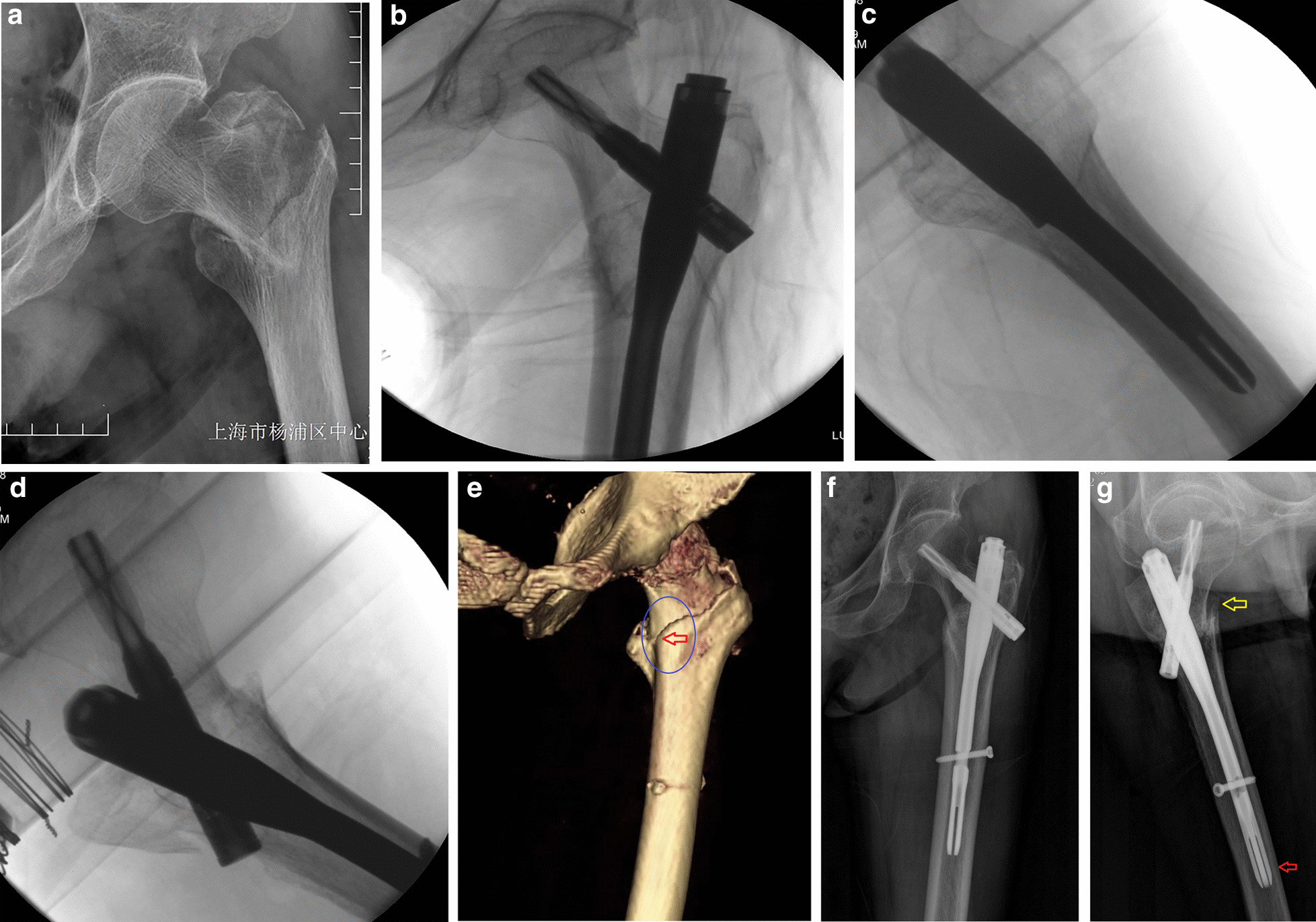
An 87-year-old woman with trochanteric femur fracture that treated by closed reduction and cephalomedullary nail fixation. **a** The fracture type was classified as 2018 AO/OTA 31A1.2. **b**–**d** Immediate postoperative fluoroscopy in AP, lateral, and anteromedial oblique views showed a good reduction quality, with smooth anteromedial cortex apposition (neutral relation). **e** Postoperative 3D CT image showed loss of cortical contact at anteromedial inferior corner. The proximal neck was sagged posteriorly into the distal medullary canal (the red arrow). **f**, **g** Follow-up X-ray in 6 months. AP view showed the backout of the helical blade by over-sliding, and lateral view showed loss of cortical contact of the anterior cortex with posterior sagging. The red arrow indicates anterior swing of the nail; the yellow arrow indicates negative anteromedial cortex relation, thus a sagittal pendulum-like movement was happened with lower filing ratio of distal nail/canal diameter

However, the status of anteromedial cortical apposition in immediate postoperative fluoroscopy is not always in accordance with postoperative 3D CT images after head–neck sliding [12]. Generally, approximately 30% of cases lose the cortical contacts at the anteromedial corner and classified as being negative in follow-ups [12, 13] (Fig. 1); the loss are usually accompanied by over-sliding, femoral neck shortening and varus, and lower functional outcomes [9, 14]. Thus, the current study aimed to identify which factors could predict the postoperative loss of anteromedial cortical contact (negative) after cephalomedullary nailing.

## Methods

### Patients and inclusion criteria

After Institutional Review Board approval, all patients who sustained trochanteric hip fractures from January 2017 to December 2019 were retrospectively reviewed. The inclusion criteria were as follows: (1) age at or over 60 years, (2) fresh trochanteric femur fractures, (3) AO/OTA-2018 classifications 31A1 and A2 [15, 16], (4) treated with intramedullary nail fixation, and (5) complete set of preoperative radiographs, intraoperative fluoroscopies, and postoperative CT scanning and 3D reconstructions, as well as having at least six months of postoperative follow-up images. The exclusion criteria were as follows: (1) 31A3 trochanteric fractures as this pattern has reversed fracture direction to the intertrochanteric line from greater to lesser trochanter, and (2) pathological fractures.

Patients were grouped according to the status of the full-range observed anteromedial cortex reduction in 3D CT as contact “yes” (Group 1, *n* = 84) and contact “no” (Group 2, *n* = 38). All of the radiographic measurements were evaluated in both groups. Patient demographic data and clinical characteristics were obtained from the medical records, including age, sex, side, American Society of Anaesthesiologists (ASA) score, and the severity of osteoporosis at the time of fracture. Osteoporosis was evaluated by using the contralateral radiograph according to the Singh Index.

### Treatment protocol

All patients had the same treatment protocol, fixed by cephalomedullary nails (Proximal Femoral Nail Anti-rotation-II, PFNA-II). In this study, all the cases were fixed with a cephalomedullary nail with distal diameter of 10 mm. All of the procedures were performed with patients in the supine position on a fracture table under spinal or general anesthesia. Routine closed reduction maneuvers, including abduction, traction, and internal rotation were performed to obtain fracture alignment and confirmed by fluoroscopy in AP, lateral, and anteromedial oblique views. If closed reduction was not possible, especially in the lateral sagittal view, for example, head–neck posterior sag or displacement, intraoperative instrument manipulation (usually with the use of a bone hook) was performed through the lateral incision from the helical blade entry route. We did not accept any negative cortical apposition in either AP or lateral fluoroscopies before nailing. However, if re-displacement of fracture reduction occurred during the nailing process (usually in the lateral view), especially the helical blade had been hammered in, we accepted it and no further manipulation was attempted, especially in older frail patients [12].

### Rehabilitation and follow-up

For rehabilitation after surgery, we have a protocol by combined evaluation of the postoperative radiographic stability score [17] and the patient’s physical capability and willingness. If it was judged as good (8 points) in stability score and patients’ capability, early weight-bearing standing and walking were encouraged within one week; otherwise, bed rest was recommended for one month, with no sitting and turning restrictions.

The follow-up time was set as at least six months, which was regarded as either fracture union or fixation failure [18, 19]. Our primary outcome included changes in fracture reduction in the anteromedial cortex. The secondary outcomes included healing and mechanical complications, including varus collapse, over-sliding, non-union, cut-out, cut-through, implant failure, and reoperation. Varus collapse was defined as NSA reduced by > 10°. Over-sliding was defined as helical blade lateral telescoping of ≥ 10 mm [20, 21].

### Parameters measurement

As standard, three immediate postoperative fluoroscopies were obtained with the patient in fracture table after surgery, i.e., AP, lateral (axial), and 30-degree anteromedial oblique views. We measured fracture reduction quality [8], NSA, TAD [22], Cal-TAD [23], Parker ratio [24] (estimation of the blade position within the femoral head), and the distal nail filling ratio. For the measurements, the known proximal diameter of the nail was used to correct the image magnification.

Subsequently, the fracture reduction quality score was assessed according to the four-point method [8]. Combined with Garden alignment and anteromedial cortical relation in AP and lateral fluoroscopies, fracture reduction quality was classified as being good (4 points), acceptable (3 points), or poor (2 points and below) (Table 1).

**Table 1:** Quality of fracture reduction in unstable trochanteric hip fractures

Items	Scores
*Garden alignment*	
AP view: normal or slight valgus^※^	1
Lateral view: angulation < 20°	1
*Fragment displacement of head–neck*	
AP view: positive or neutral medial cortex apposition	1
Lateral view: smooth continuity of anterior cortex	1
*Quality of fracture reduction*	
Good	4
Acceptable	3
poor	≤ 2

^※^Slight valgus means the valgus angle of no more than 10°

**Fig. 2:**
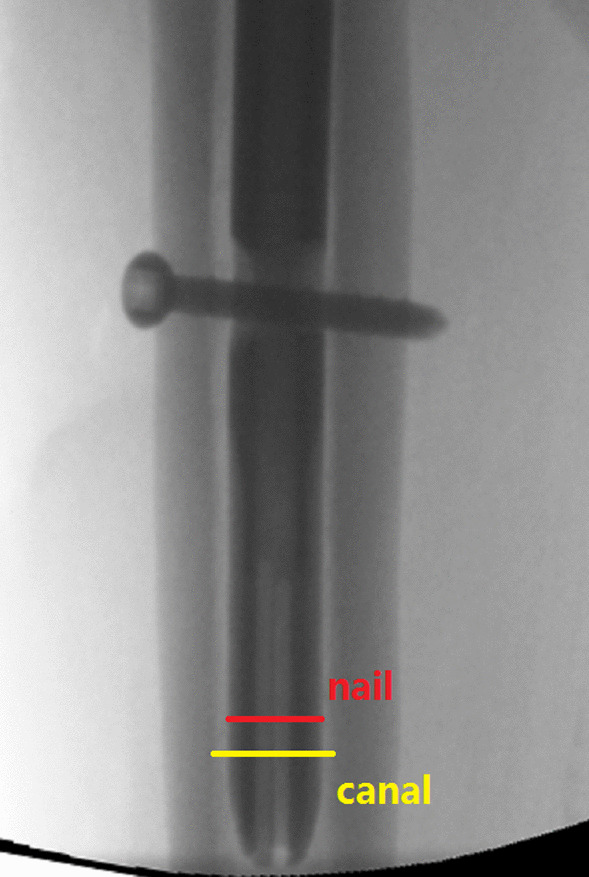
AP filling ratio was calculated in fluoroscopic image by the pixels of the distal nail (known as 10 mm, measured at 1 cm above the tip of the nail) to the pixels of the corresponding medullary canal diameter

**Fig. 3:**
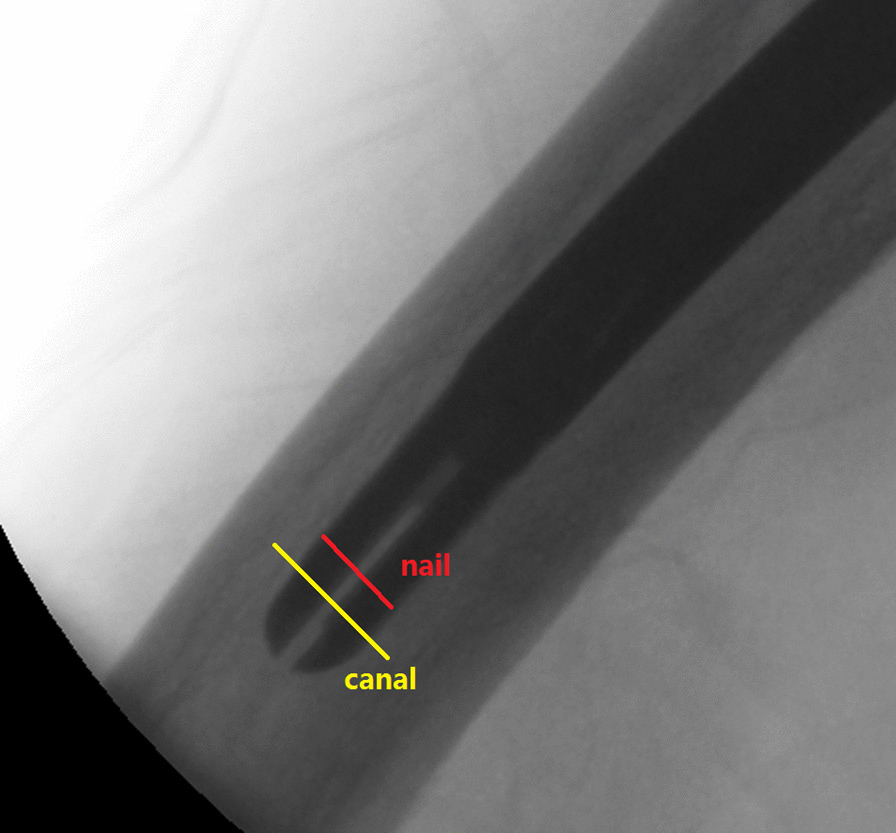
Lateral filling ratio was measured and calculated in fluoroscopic image

The filling ratio of the distal nail segment to the medullary canal was a new parameter and defined as the occupied proportion of the nail diameter to the inner medullary canal. Using Adobe Photoshop CC 2018 software, this variable was measured in standard AP (Fig. 2) and lateral fluoroscopies (Fig. 3) using the pixel ruler technique.

The images from 3D CT were considered to be accurate and standard for fracture reduction assessments, as they can be rotated 360° to provide a full range view of the relationship between the head–neck fragment and the femoral shaft. The statistical analysis was performed based on the mean value of the data measured by the two gaugers. If divergence was encountered, we obtained a conclusion via group discussion with a senior professor.

### Statistical analysis

SPSS 22.0 software (SPSS Inc., Chicago, IL, USA) was used for the statistical analyses. Continuous data were presented as means and standard deviation (SD). Categorical data were presented as counts and percentages. The univariate logistic regression analysis was performed to assess the risk factors for the loss of anteromedial cortical support. Variables with a *P*-value < 0.10 were further included in the multivariate analysis. A *P*-value less than 0.05 was defined as being statistically significant. The receiving operating characteristic (ROC) curve for distal nail filling ratio in lateral fluoroscopy was used to calculate the area under the curve. The best threshold value of the distal nail filling ratio in lateral fluoroscopy was used to predict loss of anteromedial cortical support.

## Results

There were 34 males (28% of the sample) and 88 females (72% of the sample), with an average age of 83.0 years. The right side was affected in 63 patients (52%), and the left side was involved in 59 (48%). The AO/OTA-2018 classification identified 45 cases (37%) as type A1 and 77 cases (63%) as type A2.

Of the 122 patients included in this study, all patients had a positive or neutral cortex-to-cortex reduction in the AP and lateral immediate postoperative fluoroscopic views. The fracture reduction quality score was 4 points (good) in 87 cases (71%) and 3 points (acceptable) in 35 patients (29%). The 1-point loss in most cases was due to the residual gap between the head–neck fragment and shaft, which was larger than one cortex thickness. Finally, postoperative 3D CT full-range images revealed that 84 cases (69%) were categorized as having true anteromedial cortex contact (positive or anatomic, Group 1), and 38 cases (31%) were categorized as having lost contact (negative, Group 2).

### Univariate and multivariate analysis

From the univariate logistic regression analysis, no significant differences were found concerning age, sex, side injured, ASA score, Singh index, helical blade TAD, Cal-TAD, NSA, and Parker ratio in the AP view. There were significant differences in AO/OTA classification (*P* = 0.045), reduction quality score (*P* < 0.001), Parker ratio in the lateral view (*P* = 0.012), nail filling ratio in the AP (*P* < 0.001) and lateral views (*P* < 0.001). Furthermore, in the multivariate analysis, only the distal nail filling ratio in the lateral view (*P* < 0.001) and fracture reduction quality score (*P* < 0.001) were considered to be significant predictive factors for the postoperative loss of anteromedial cortex contact (Table 2).

**Table 2:** Logistic regression model to detect possible risk factors for losing cortex-to-cortex support

Factors	All	Group 1 (*n* = 84)	Group 2 (*n* = 38)	Univariate analysis		Multivariate analysis	
		Contact	No contact	OR (95% CI)	*P* value	Adjusted OR (95% CI)	*P* value
*Side*				0.909 (0.422–1.957)	0.807		
Left	59 (48)	40 (48)	19 (50)				
Right	63 (52)	44 (52)	19 (50)				
*Gender*				1.681 (0.679–4.163)	0.262		
Male	34 (28)	26 (31)	8 (21)				
Female	88 (72)	58 (69)	30 (79)				
Ages (mean ± SD)	83.0 ± 8.2	82.3 ± 8.7	84.6 ± 7.0	1.037(0.986–1.091)	0.155		
ASA Score 1/2/3/4	3/50/69/0	1/35/48/0	2/15/21/0	0.820 (0.409–1.646)	0.577		
Singh Osteoporosis index I/II/III/IV/V/VI	4/5/29/50/24/10	2/4/18/35/15/10	2/1/11/15/9/0	0.782 (0.550–1.111)	0.170		
*AO classification*				2.417 (1.019–5.732)	**0.045**	0.961 (0.217–4.255)	0.958
31.A1	45 (37)	36 (43)	9 (24)				
31.A2	77 (63)	48 (57)	29 (76)				
*Reduction quality*				0.043 (0.016–0.118)	** < 0.001**	0.072(0.017–0.307)	** < 0.001**
Good	87 (71)	76 (91)	11 (29)				
Acceptable	35 (29)	8 (9)	27 (71)				
NSA (mean ± SD)	130.7 ± 5.3	130.6 ± 5.4	130.7 ± 5.1	1.003 (0.933–1.079)	0.930		
TAD (mean ± SD)	22.5 ± 4.5	22.8 ± 4.6	21.9 ± 4.2	0.958 (0.879–1.044)	0.331		
Cal-TAD (mean ± SD)	23.3 ± 4.6	23.4 ± 4.7	23.2 ± 4.5	0.993 (0.914–1.079)	0.865		
FR in AP view (mean ± SD)	66.9 ± 10.9	70.8 ± 9.6	58.4 ± 8.6	0.868 (0.821–0.917)	** < 0.001**	0.936 (0.862–1.017)	0.121
FR in Lat view (mean ± SD)	58.3 ± 10.7	63.1 ± 8.3	47.8 ± 7.1	0.761 (0.688–0.843)	** < 0.001**	0.793 (0.701–0.896)	** < 0.001**
Parker ratio in AP view (mean ± SD)	42.1 ± 8.1	41.5 ± 7.8	43.5 ± 8.7	1.030 (0.982–1.081)	0.222		
Parker ratio in Lat view (mean ± SD)	46.4 ± 8.9	47.8 ± 8.7	43.4 ± 8.6	0.942 (0.900–0.987)	**0.012**	0.959 (0.887–1.037)	0.293

Values with statistically significant difference are marked in bold

NSA, Neck Shaft angle; TAD, Tip-apex distance; FR, filling ratio; AP, anteroposterior; Lat, lateral; SD, standard deviation

### Mechanical complication

No patient exhibited cut-out, cut-in, fracture non-union, or nail broken characteristics for the follow-up analysis. The mechanical complication details of varus collapse and excessive lateral sliding are shown in Table 3.

**Table 3:** Mechanical complications

Mechanical complications	Group 1 No. of cases (%)	Group 2 No. of cases (%)	*P* value
Varus collapse (> 10°)	2 (2.4)	8 (21.1)	**0.001**
Excessive lateral sliding (≥ 10 mm)	1 (1.2)	6 (15.8)	**0.004**

Values with statistically significant difference are marked in bold

### ROC curve predicting cortex contact change

**Fig. 4:**
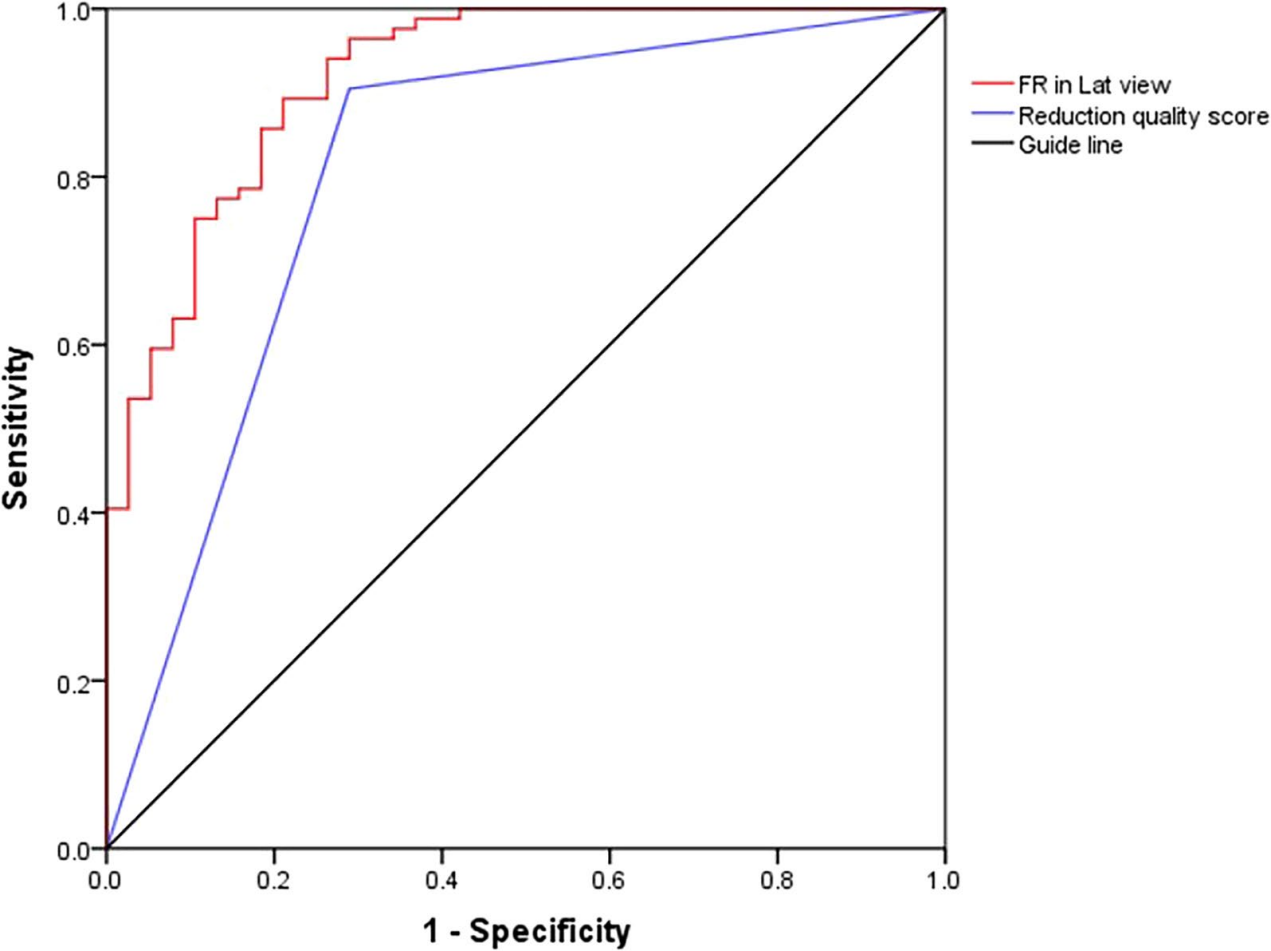
Area under the curve (AUC) was 0.919 for the filling ratio of the distal segment to medullary canal on the lateral view (95% confidence interval 0.867 to 0.972) and 0.808 for the reduction quality score (95% confidence interval 0.714 to 0.901). The recommended lateral filling ratio of the distal nail segment to medullary canal for balancing sensitivity and specificity was 53 (%)

The ROC curve was used to estimate a threshold value that can predict losing of anteromedial cortical support. The criterion for alteration was 53% for filling ratio of the distal nail segment to the medullary canal in the lateral view for balancing sensitivity (89.3%) and specificity (78.9%) (Fig. 4).The area under the curve (AUC) was 0.919 for the filling ratio in the lateral view and 0.808 for the reduction quality.

## Discussion

Our study enrolled 122 geriatric trochanteric hip fractures fixed with short trochanteric femoral nails. All patients had a positive or neutral anteromedial cortex reduction in the AP and lateral views on immediate postoperative fluoroscopy. Postoperative 3D CT full-range images revealed that 38 cases (31%) lost their cortical contact. In both the univariate and multivariate analyses, the fracture reduction quality score and decreasing distal nail filling ratio in the lateral view were significant risk factors for predicting the loss of anteromedial cortical contact.

### Risk factors for complication and failure

Five major factors have been recognized as predisposing fixation complications and treatment failure, including bone quality, fracture morphology, implant selection, implant placement, and, most importantly, fracture reduction quality [7]. However, of these five factors, only three factors can be controlled by the surgeon. For implant selection, more surgeons now prefer the use of intramedullary nails [25–27]. For the position of the helical blade in the femoral head, the TAD, Cal-TAD, and Parker ratios provide good guidance and are practiced in daily work [3, 28–30]. For the quality of fracture reduction, there has been a recent tendency for surgeons to pay more attention to the anteromedial cortex rather than to the posteromedial lesser trochanter, as has been previously described [31]. In addition, Kim et al. [32] showed that greater trochanter separation was a risk factor for posterior sagging, which was caused by the instability of the posterior coronal fragment. Parry et al. [33] demonstrated that calcar gapping and fracture classification were related to over-sliding of lag screws. Ciufo et al. [34] confirmed that lateral wall fracture, neck-shaft malreduction, and residual basicervical gapping were related to lag screw cut-out. Our study concluded that the fracture reduction score and the filling ratio of the distal nail segment to the medullary canal in the lateral view are closely associated with the anteromedial cortex buttress alteration. Therefore, in addition to fracture reduction with anteromedial cortex support, we also recommend that surgeons choose a nail diameter as thick as possible, provided that it can be manually inserted without femoral shaft reaming.

### Anteromedial cortical support reduction

It is well-known that fracture reduction quality is of paramount importance and is the first prerequisite factor for the successful treatment of trochanteric femur fractures [35]. Anteromedial cortical support reduction provides mechanical stability to share loads from the implant and biological environments in fracture healing [13, 36]. However, redisplacement of the head–neck fragment after cephalomedullary nailing is still a common phenomenon, which leads to the transition of primarily good/acceptable fracture reduction quality changes into a poor category and causes varus deformity, femoral neck shortening, healing complications, or even implant failure [8, 37].

Anteromedial cortex-to-cortex support reduction was first introduced in 2015 by Chang and colleagues for unstable pertrochanteric femur fractures [8]. It involves a pattern of non-anatomical functional buttress reduction. It is specific for the proximal femur, as it relates to the NSA, and when various implant devices with sliding mechanisms are used for fixation. Controlled fracture impaction via limited telescoping provides secondary axial and torsional stability between the head–neck fragment and the femur shaft [38].

A full description of anteromedial cortical support reduction or cortex-to-cortex contact involves the assessment in three fluoroscopic views, i.e., the AP view (for the medial cortex), the lateral view (for the anterior cortex), and the 30-degree oblique view (for the anteromedial inferior corner cortices) [39]. The relationship between the head–neck fragment and the femoral shaft, which describes the position of their cortical layers or the trend in their changes of position after sliding along the implant axis (usually by 130°), is evaluated and classified into three categories (positive, neutral, and negative) for both medial and anterior cortical appositions.

We do not accept any negative cortical relations in AP, lateral, or oblique fluoroscopic views before cephalomedullary nailing in clinical practice. Re-manipulation is performed by using a closed maneuver or inserted instruments (such as a bone hook) to dig out the head–neck from its intramedullary position. However, redisplacement may occur during surgery, and there is usually no opportunity for readjustments in older frail patients after nailing.

### Predictors for loss of anteromedial cortex reduction

Anteromedial cortical support is favorable for secondary stability after limited lateral sliding between head–neck fragments to the femoral shaft. However, several factors may interfere with sliding movement and change the final cortical apposition at the anteromedial inferior corner. These factors include (but are not limited to) the bearing and residual space between the head–neck fragment and femoral shaft, the ability to initiate head–neck sliding, the direction of sliding, the rotation and/or tilting during sliding, the external rotation of the femoral shaft (which may open a gap and step between the two anterior cortices), and the pendulum-like movement of the nail in the medullary canal [37].

The residual space between the head–neck and femoral shaft in the medial and/or lateral cortices is a major predictive factor. Typically, one cortex thickness is used as the criterion, and a residual gap/space larger than one cortex thickness (especially in the anterior region) is considered a significant risk factor. As the head–neck fragment obliquely slides in the inferior lateral direction, a larger gap likely predicts the loss of cortical contact. In this cohort study of patients who were evaluated as possessing acceptable fracture reduction quality (i.e., 3 points), the loss of 1 point in most cases was due to the larger residual gap/space between the anterior cortices in lateral fluoroscopy.

Pendulum-like movement in short nails is another risk factor. The distal interlocking screw acts as a pivotal point, leading to the proximal nail with the head–neck fragment toggling back and forth in both coronal (AP view) and sagittal (lateral view) directions. The amplitude of the proximal movement is determined by the tip segment length distal to the locking screw, the width of the medullary canal, and whether there is a posterior coronal fragment involving the lesser trochanter and posteromedial cortex. In the clinic, this phenomenon is especially prominent for short nails with significantly shorter nail tip lengths distal to the interlocking screw and with a larger posterior banana-like fragment, which consists of the posterior coronal components of the greater trochanter, the posterior crest, the lesser trochanter, and the posteromedial cortex. Chang et al. [17] described this phenomenon and recommended using full-length nails to prevent this deformity.

**Fig. 5:**
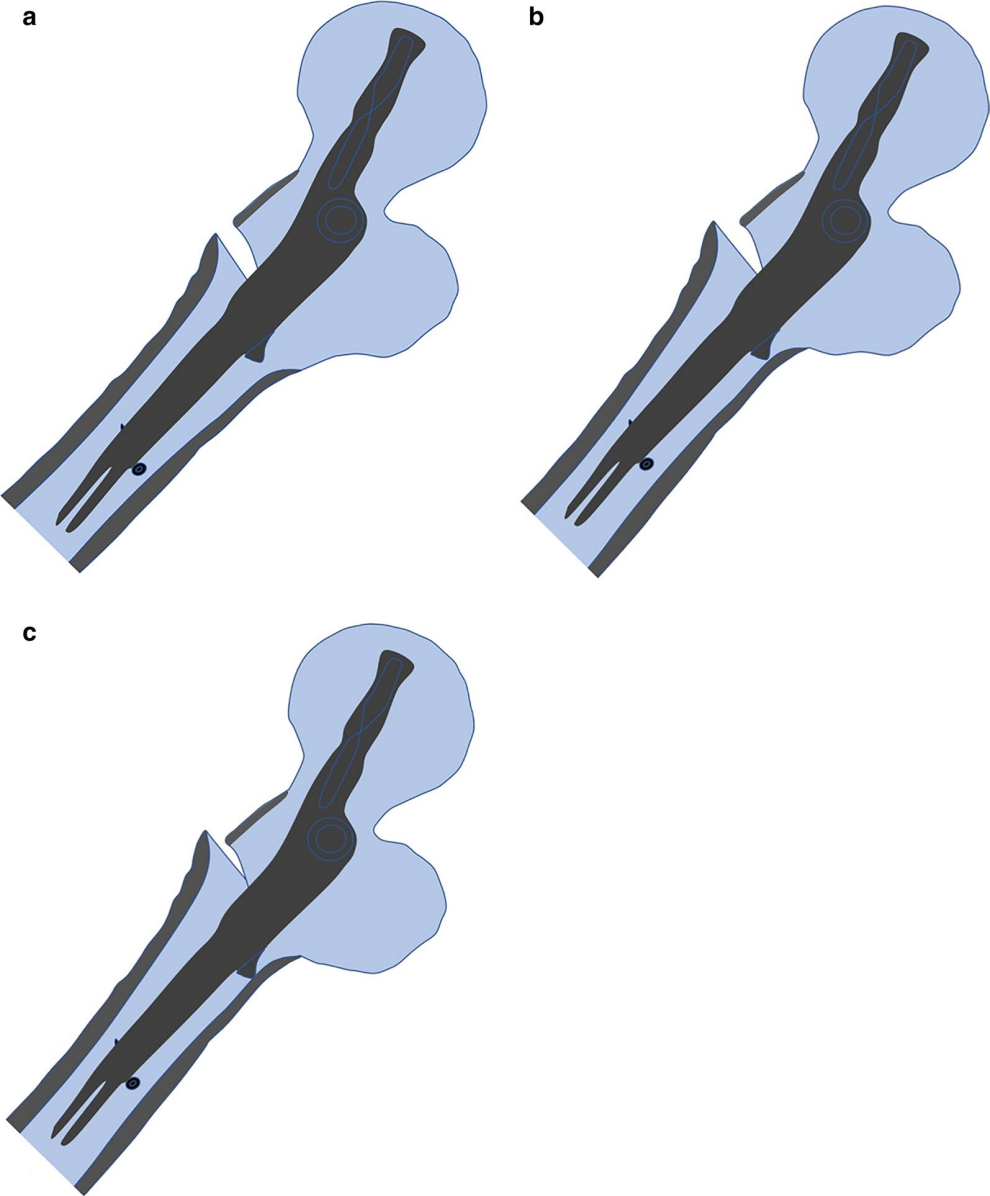
Schematic drawing to show loss of cortex to cortex apposition with low filling ratio in lateral view. **a** Smooth anterior cortical relation after nailing fixation. **b** Pendulum-like movement in sagittal plane leads to the proximal nail and head–neck fragment shifts backward, and results in a posterior sag of the anterior cortex. **c** The head–neck fragment loses its cortical buttress from the femoral shaft during sliding

As the proximal femoral shaft canal diameter in the sagittal plane is usually larger than that in the coronal plane, the distal nail filling ratio is inversely smaller in the sagittal plane than in the coronal plane, which indicates that pendulum-like movement may occur more frequently and severely in the sagittal plane and result in the proximal nail and head–neck fragment shifting backward (Fig. 5). Clinically, this phenomenon may occur with the following two conditions: (1) detachments of the lesser trochanter and posteromedial cortex (A2.3 in 2018-AO/OTA classification) [40], whether it occurs in a large banana-like posterior coronal fragment or a comminuted pattern [16], and (2) a lower filling ratio of the distal nail to a capacious medullary canal. We referred to the pendulous wave as a swing tail effect.

Recently, several studies have focused on the effects of distal nail diameter. Cheung et al. [41] studied the distal nail diameter (10 mm vs. 11 mm) in short nails and found no differences in the postoperative complications. In a biomechanical study, Ceynowa et al. [42] reported that the large discrepancy in the diameters of the medullary canal (reamed to 18 mm) and the distal part of the nail (11 mm) would result in the tip of the nail migrating laterally as far as causing an impingement to the lateral cortex. However, these authors did not consider the filling ratio concept. Durusoy et al. [43] revealed that the nail diameter made a difference in intramedullary nail movement by using biomechanical tests. A low filling ratio has been associated with varus collapse progress and cut-out complications. However, the research has only explored the match between the diameter of the nail and the intramedullary canal in the coronal plane. The clinical influence of the filling ratio of the nail in the sagittal plane has not been previously reported.

In our opinion, suffusion of the distal nail to the medullary space will enhance the stability by preventing pendulum-like movement, especially in the sagittal plane. It will contribute to the overall stability of the fracture that is fixed with the intramedullary nail. Therefore, the negative transformation of the support status would not be easy to trigger. In addition, two distal screws would also be a benefit for preventing the pendulum-like movement. Distal stability combined with anteromedial contact would be beneficial for reducing the incidence of lateral sliding and varus collapse, as was observed in our follow-up analysis.

## Study limitations

There were several limitations of this study. First, it was limited by the retrospective analysis itself. Second, the CT examination was not regularly performed for all the patients, the sample of enrolled patients was small. Additionally, 3D CT was deemed to be the standard in judging anteromedial cortex-to-cortex contact. Therefore, patients who lacked CT data were excluded. We also recognized that the filling ratio of the distal nail in the medullary space only plays a limited role in intramedullary nail fixation. Further studies with large samples and prospective design with clinical follow-up data would be beneficial for demonstrating more reliable conclusions.

## Conclusions

In conclusion, for trochanteric femur fractures treated with intramedullary nails, the filling ratio of the distal nail to the medullary canal in the lateral view plays a role in predicting the maintenance of anteromedial cortex-to-cortex supports. A filling ratio over 50% on the lateral view may be regarded as the threshold for preventing pendulum-like movement of the nail in the medullary canal and for eliciting a benefit in mechanical complications. Our conclusions remind us of the existing possible fixation impact of the nail diameter matching the medullary space. It is recommended that thicker nails be used without femoral shaft reaming.

## Data Availability

The datasets used and analyzed during the current study are available from the corresponding author on reasonable request.

## Abbreviations

TAD: Tip-apex distance
Cal-TAD: Calcar-referenced TAD
NSA: Neck-shaft angle
AP: Anteroposterior
ASA: American Society of Anaesthesiologists
ROC: Receiver operating characteristic
AUC: Area under the curve
